# Effect of different CT scanners and settings on femoral failure loads calculated by finite element models

**DOI:** 10.1002/jor.23890

**Published:** 2018-04-20

**Authors:** Florieke Eggermont, Loes C. Derikx, Jeffrey Free, Ruud van Leeuwen, Yvette M. van der Linden, Nico Verdonschot, Esther Tanck

**Affiliations:** ^1^ 611 Orthopaedic Research Laboratory Radboud Institute for Health Sciences Radboud university medical center P.O. Box 9101 Nijmegen 6500 HB The Netherlands; ^2^ Radiotherapeutic Institute Friesland Leeuwarden The Netherlands; ^3^ Department of Radiation Oncology University Medical Center Groningen University of Groningen Groningen The Netherlands; ^4^ Department of Radiotherapy Radboud university medical center Nijmegen The Netherlands; ^5^ Department of Radiotherapy Leiden University Medical Center Leiden The Netherlands; ^6^ Laboratory of Biomechanical Engineering University of Twente Enschede The Netherlands

**Keywords:** CT scanner, CT protocol, finite element model, femur, failure load

## Abstract

In a multi‐center patient study, using different CT scanners, CT‐based finite element (FE) models are utilized to calculate failure loads of femora with metastases. Previous studies showed that using different CT scanners can result in different outcomes. This study aims to quantify the effects of (i) different CT scanners; (ii) different CT protocols with variations in slice thickness, field of view (FOV), and reconstruction kernel; and (iii) air between calibration phantom and patient, on Hounsfield Units (HU), bone mineral density (BMD), and FE failure load. Six cadaveric femora were scanned on four CT scanners. Scans were made with multiple CT protocols and with or without an air gap between the body model and calibration phantom. HU and calibrated BMD were determined in cortical and trabecular regions of interest. Non‐linear isotropic FE models were constructed to calculate failure load. Mean differences between CT scanners varied up to 7% in cortical HU, 6% in trabecular HU, 6% in cortical BMD, 12% in trabecular BMD, and 17% in failure load. Changes in slice thickness and FOV had little effect (≤4%), while reconstruction kernels had a larger effect on HU (16%), BMD (17%), and failure load (9%). Air between the body model and calibration phantom slightly decreased the HU, BMD, and failure loads (≤8%). In conclusion, this study showed that quantitative analysis of CT images acquired with different CT scanners, and particularly reconstruction kernels, can induce relatively large differences in HU, BMD, and failure loads. Additionally, if possible, air artifacts should be avoided. © 2018 Orthopaedic Research Society. © 2018 The Authors. *Journal of Orthopaedic Research*® Published by Wiley Periodicals, Inc. on behalf of the Orthopaedic Research Society. J Orthop Res 36:2288–2295, 2018.

Bone metastases in patients with advanced cancer are very common. These metastases cause pain and induce a certain risk of pathological fracture. Predicting the fracture risk is important for deciding on treatment strategy, since patients with a low fracture risk are conservatively treated with local radiotherapy to relieve pain, whereas patients with a high fracture risk undergo stabilizing prophylactic surgery.^1^, ^2^ However, in present clinical practice, it appears to be difficult to distinguish between low and high fracture risk patients, causing a large number of over‐ and undertreated patients.^1^

Subject‐specific finite element (FE) models are a promising tool in calculating strength of femora with (artificial) metastatic lesions. Experimentally, these FE models have shown promising results for calculation of fracture risk.^3^, ^4^, ^5^ For such FE models, quantitative computed tomography (QCT) scans are used to segment the subject‐specific femur geometry. Also, bone mineral density (BMD) is often calculated with the use of a calibration phantom under the patient. The subject‐specific geometry and BMD are used as input for the FE models. Recently, studies using FE models showed promising results in discriminating patients with a low fracture risk from patients with a high fracture risk.^6^, ^7^

However, it has been shown that scanning the same subject using comparable protocols on different CT scanners can result in different Hounsfield Units (HU).^8^, ^9^, ^10^, ^11^ When comparing high versus low resolution CT scans, Dragomir‐Daescu et al.^12^ showed that there were differences in FE strength in a fall configuration of maximally 1,500 N (∼45%). Another study showed that the FE calculated failure load could differ up to 2,500 N (∼23%) when simulating a single‐leg stance induced fracture based on CT scans of a healthy subject scanned on two different CT scanners.^13^ The authors additionally showed that accounting for these inter‐scanner differences is difficult.

Another problem arises when using clinical CT scans as input for the FE models. Potential changes in CT settings by deviating from a standard protocol may influence HU and subsequently the outcome of FE models.^14^ Additionally, it appears that air between calibration phantom and patient induces an artifact in the calibration phantom. Such air gaps are common when scanning cancer patients, as patients’ knees are often placed on a cushion to relieve pain. These air artifacts have been described before,^11^, ^15^ although it is unclear how it affects the calibration to in vivo BMD values and subsequent calculation of failure load.

Since we are currently performing a multicenter patient study for in vivo validation of our FE models, we want to unravel these problems. As a first step, the effect of different CT scanners and CT protocols on HU and BMD using tissue characterization phantoms was recently investigated.^11^ We found differences between CT scanners in HU in bone‐equivalent regions within the phantom up to 10%, and these differences decreased to maximally 7% when HU were calibrated to BMD via a calibration phantom under the tissue characterization phantom. Additionally, variations in CT settings, mainly reconstruction kernel, resulted in differences in bone‐equivalent HU up to 16%. Also, air between calibration phantom and tissue characterization phantom affected the calibration. These effects were scanner‐dependent.

The next step is to determine how differences in CT equipment or protocols affect HU and BMD in a more physiological setting, for example, when scanning femoral tissue. In that case, FE failure loads can be calculated as well. Femoral tissue is more heterogeneous than inserts in a phantom, and other beam hardening and partial volume effects can be expected under physiological circumstances. Therefore, the aims of this study were to quantify the effect of (i) different CT scanners; (ii) different CT protocols (with variations in slice thickness, field of view (FOV), and reconstruction kernel); and (iii) air between calibration phantom and patient, on HU, BMD, and FE failure load.

## METHODS

### Cadaveric Femora

Six fresh frozen femora (three male, three female; mean age 86.7 years, range 82–95 years) were obtained from the Anatomy department of the Radboud university medical center. Soft tissue was removed and the proximal femora were cut at 24 cm. All femora were embedded distally in polymethylmethacrylate according to previously described protocol.^3^

### QCT Scanning

Four radiotherapy institutes participated in this study (Radboud university medical center Nijmegen, Leiden University Medical Center, Radiotherapeutic Institute Friesland Leeuwarden, Bernard Verbeeten Institute Tilburg). These institutes used CT scanners of three manufacturers (Philips Brilliance Big Bore (Philips Medical Systems, Eindhoven, The Netherlands, two institutes), GE Optima CT580 (GE Healthcare, Milwaukee, WI, USA) and Toshiba Aquilion/LB (Toshiba Medical Systems, Tokyo, Japan), abbreviated with P1, P2, GE, and To, respectively).

The femora were placed in an anatomically shaped model of the lower body,^16^ mimicking the human body. This body model was positioned in the isocenter of the CT scanner atop a solid calibration phantom (Image Analysis, Columbia, KY), containing four known calcium hydroxyapatite concentrations (0, 50, 100, and 200 mg/cm^3^ CaHA). The known densities in this phantom were used to calibrate HU to CaHA density, which is a measure of BMD.

Standard scans were acquired according to the standard patient study protocol, using the following settings: 120 kV, variable mA (calculated by the scanner software), 1 s rotation time, 3 mm slice thickness, FOV 480 mm, in plane resolution 0.9375 mm, standard reconstruction kernel (B on P1 and P2, standard on GE, and FC17 on To), pitch <1. To study the effect of different CT settings, the femora were scanned using the standard protocol, and with variations in slice thickness (1 mm), FOV (550 mm, in plane resolution 1.0742 mm), and reconstruction kernel (detailed: D on P1, UB on P2, detail on GE, and FC43 on To),^11^ which were the most commonly applied deviations from the standard protocol in our patient study. All combinations were applied, resulting in a total of eight scans with different settings per CT scanner. Subsequently, the effect of an air gap between calibration phantom and lower body model was assessed by lifting the knees of the lower body model 5 and 10 cm, respectively. The latter scans were acquired on every CT scanner using the standard protocol, and mimicked a patient's knees being supported by a cushion.

### Cortical and Trabecular ROI

**Figure 1:**
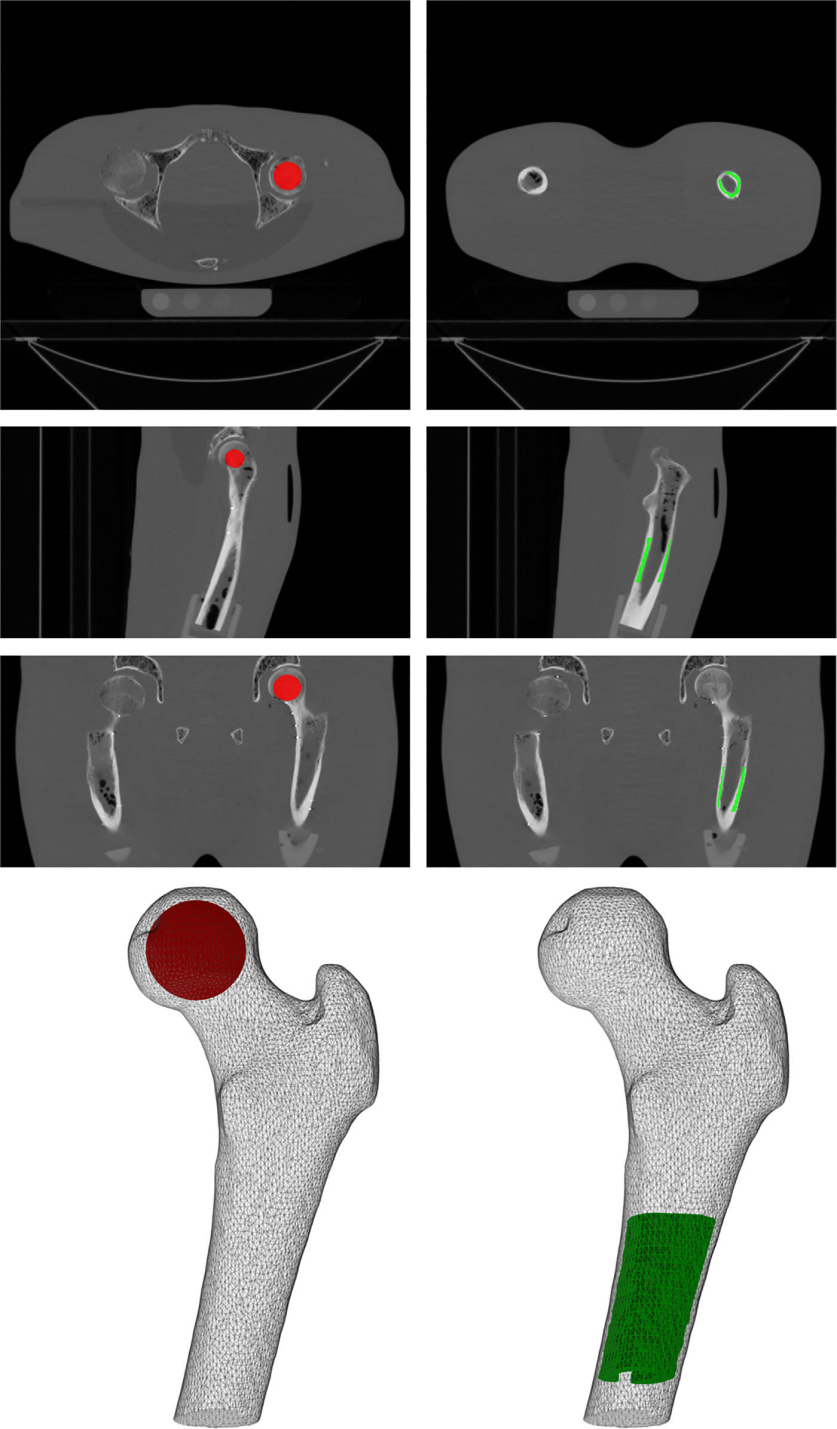
Axial, sagittal, and coronal slice and 3D plots of the trabecular (red) and cortical (green) ROIs.

Subject‐specific femoral geometry was obtained from the standard CT scans (3 mm, FOV 480, standard reconstruction kernel) by selecting the voxels containing femoral tissue in each slice (Mimics 14.0, Materialise, Leuven, Belgium). For all other scans, the femur geometry was registered to the CT scan using software containing algorithms for registration of medical images (elastix^17^, ^18^). Subsequently, a cortical and a trabecular region of interest (ROI) were drawn (Fig. 1). For the cortical ROI, 10 cm below the femoral head, voxels were selected along 6 cm of the cortex of the femoral shaft. For the trabecular ROI, 75% of the sphere that fitted the femoral head was used. ROIs were registered using the transformation of the femoral registration. Mean HU were obtained for each ROI and were calibrated to mean BMD.

**Figure 2:**
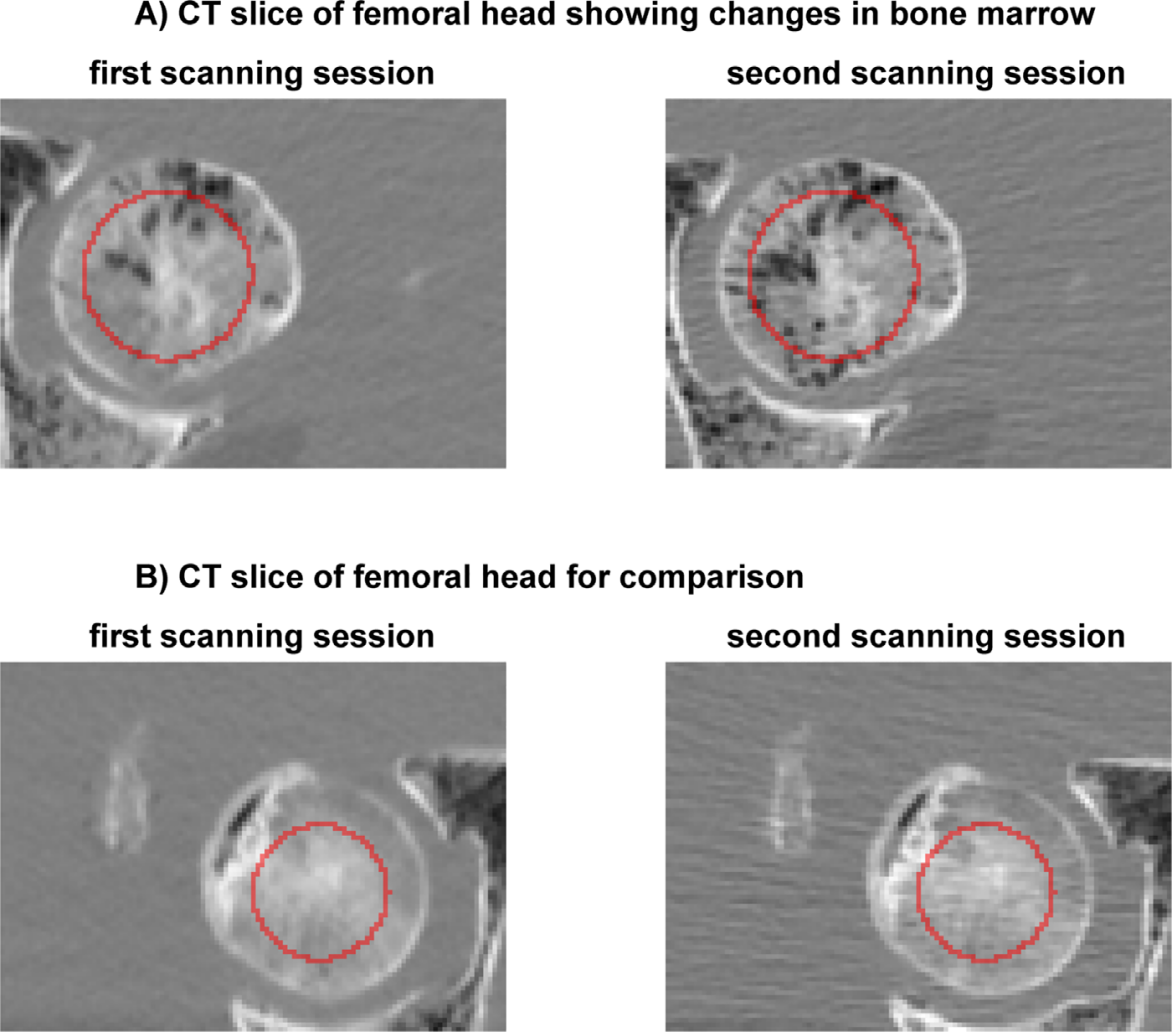
One femur seemed to have lost bone marrow in the femoral head after the first compared to the second scanning session (A). For comparison, we show another femur after the first and the second scanning session (B). The red line depicts the edge of the trabecular ROIs.

In one of the femora, for unknown reasons, bone (marrow) composition in the femoral head seemed to have changed after the first scanning session (P1), when comparing it with scans of the next scanning sessions (P2, GE, and To, Fig. 2). Therefore, this femur was excluded from analysis of the trabecular ROI. The cortical ROI was not affected. The femur was not excluded from FE analysis (see next paragraph), as in most part of the femoral head post‐yield material behavior was not implemented in the FE models, and hence, we expect the FE failure load not to be significantly affected.

### FE Models

FE models based on the standard CT scan of every femur were constructed as described previously.^3^ The femoral geometry was converted into a solid mesh (average element volume 1.4 mm^3^, Patran 2011, MSC Software Corporation, Santa Ana, CA). Using the calibration phantom, the HU of each element were calibrated to BMD. The BMD values were subsequently converted to non‐linear isotropic bone material properties.^19^ Two bundles of high‐stiffness springs served as distal fixation and a displacement‐driven load was applied on the femoral head via a simulated cup. To prevent artifacts as a result of the loading configuration, post‐yield material behavior was not implemented in a region underneath the cup comprising the proximal elements of two third of the femoral head, and at the distal fixation comprising distal elements of a region as high as the radius of the shaft. FE simulations were performed using MSC.MARC (v2013.1, MSC Software Corporation). Incremental displacement and contact normal forces were registered and plotted in force‐displacement curves. It was assumed that fracture occurred when maximum total reaction force was reached. At the corresponding increment, a clear fracture line of plastic elements was visible.

The femur geometry from the standard scan was registered onto the non‐standard scans in order to obtain CT scan‐specific material properties. All other aspects of the FE model, for example the geometry and alignment, were left unchanged. In this way, only the material properties differed between FE models, which enabled us to study the isolated effect of variations in CT images. This resulted in a total of 240 FE simulations (10 scans of six femora on four scanners, hence 240 calculated failure loads).

**Statistical Analysis**

Linear mixed models were used to analyze effects of different CT scanners on HU and BMD in the cortical and trabecular ROI, and on FE calculated failure load. Slice thickness, FOV, and reconstruction kernel were added to the model as fixed factors to cover the effect of changes in CT settings. A random intercept was included to disregard the variability between femora. Only the interaction between CT scanners and reconstruction kernel was added to the model, as this increased the models’ fits based on likelihood‐ratio tests. All other interactions did not increase the fits and were therefore omitted from the final models. This includes the interactions between the effects of changing slice thickness, FOV or reconstruction kernel, indicating that there was no additional effect when two or more CT settings were changed.

Furthermore, linear mixed models were created to determine the effect of air between body model and calibration phantom on the outcome variables. As random factors, CT scanner and air gap (0, 5 or 10 cm) were added to the model. Again, a random intercept was included to account for the variability between the femora. For HU and failure load, no interactions were modeled, as they did not significantly improve the fit of the model without interaction based on likelihood‐ratio tests. However, the interaction between CT scanner and air gap did significantly affect the fit of cortical and trabecular BMD, and was therefore included in these two models. *P*‐values below 0.05 were considered statistically significant. All statistical analyses were performed using Stata/SE 11.2 (StataCorp LP, College Station, TX).

As descriptive statistics, median and range of cortical and trabecular HU and BMD and failure loads of the standard scans were calculated. These standard scans served as a default scan. To quantify inter‐scanner effects, results of standard scans of P1, P2, GE, and To were expressed relative to the average of these four standard scans. The effect of variations in CT settings and air gap was quantified by normalizing the results of non‐standard scans to the scanner‐specific standard scans. Results are expressed as mean percentage ± SD.

## RESULTS

### Effect of CT Scanners

**Figure 3:**
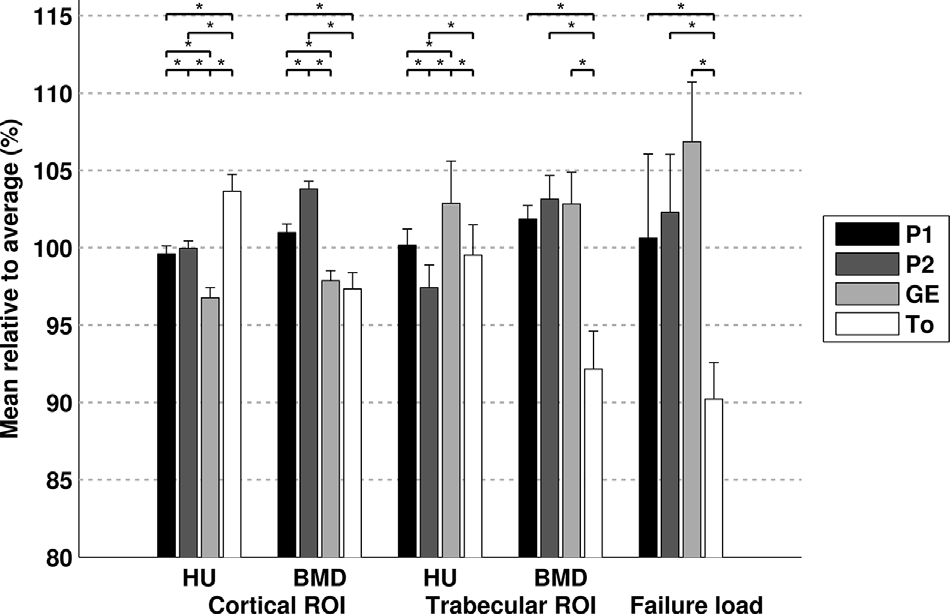
Output of the scanners using the standard protocol (3 mm slices, FOV 480, standard kernel), in % relative to the average of all scanners (mean ± SD) for HU and BMD in the cortical and trabecular ROI, and simulated failure load. *significant difference.

Medians and ranges of the standard scans for all outcome measures are depicted in Table 1. The differences in cortical HU between all CT scanners were significant (Table S‐2). Scanning on GE derived the lowest cortical HU, while scanning on Toshiba resulted in highest cortical HU, leading to a difference of on average 7 ± 2% (Fig. 3). In the trabecular ROI, lowest HU were found when scanning on P2. On the GE scanner, HU in the trabecular ROI were largest, resulting in a maximal difference between GE and P2 of 5 ± 4%.

**Table 1:** Median of the Standard Scans (3 mm Slices, FOV 480, Standard Kernel) of All Femurs for All Outcome Measures

	P1	P2	GE	To
Cortical ROI				
HU	1,150 (1,043–1,290)	1,155 (1,032–1,289)	1,118 (1,004–1,253)	1,195 (1,083–1,341)
BMD (mg/cm^3^)	1,009 (908–1,133)	1,036 (927–1,154)	978 (874–1,097)	971 (876–1,089)
Trabecular ROI				
HU	278 (103–318)	267 (99–311)	280 (108–315)	278 (98–317)
BMD (mg/cm^3^)	245 (88–276)	241 (91–281)	242 (91–272)	222 (76–254)
Failure load (*N*)	4,531 (1,272–7,152)	4,655 (1,340–7,500)	4,779 (1,499–7,224)	4,179 (1,218–6,429)

In between brackets, the range of the outcomes among the different femurs is displayed. For the outcomes of the standard scan on each CT scanner for each femur separately, see Supplementary Table S‐1.

Calibrating HU to BMD changed the differences between CT scanners (Table S‐2). In the cortical ROI, the highest BMD was found on P2, while on To BMD was the lowest, leading to a maximal variation of 6 ± 1% (Fig. 3). In the trabecular ROI, again P2 and To differed the most (11 ± 4%).

FE calculated failure load was not significantly different between P1, P2, and GE, while all differences with respect to To were significant (Table S‐2). Although not significantly different from P1 and P2, scanning on GE resulted in the highest calculated failure load, while the lowest failure load was calculated with scans of To. The maximal difference between GE and To in calculated failure load was 17 ± 5% (Fig. 3).

### Effect of CT Settings

#### Slice Thickness

**Figure 4:**
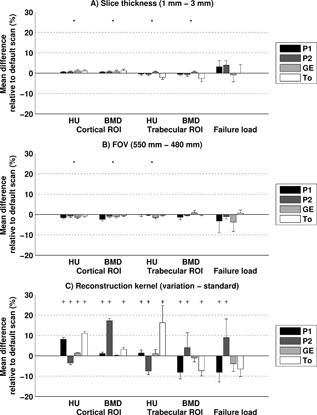
Difference between standard scan (3 mm slices, FOV 480, standard kernel) and variation as percentage of the standard scan (mean ± SD) of variations in slice thickness (A), FOV (B), and reconstruction kernel (C) for HU and BMD in the cortical and trabecular ROI, and simulated failure load. * significant effect, holds for all of the CT scanners, as the interaction between CT scanner and slice thickness or FOV was not in the statistical model. +, significant effect.

The effect of changing the standard 3 mm slice thickness to 1 mm was small (Fig. 4A, Table S‐3). Changing slice thickness resulted in maximal 1 ± 1% difference in cortical HU, 2 ± 1% in trabecular HU, 1 ± 1% in cortical BMD, and 3 ± 2% in trabecular BMD. The effect of changing the slice thickness on calculated failure load was largest on the P2 scanner, with a 4 ± 2% increase in failure load.

#### FOV

The effects of varying FOV between 480 and 550 mm were small (Fig. 4B, Table S‐3). The largest effect of variations in FOV on HU was 2 ± 0% on P1 for the cortical ROI and 1 ± 1% on GE for the trabecular ROI. After calibration, the effects were largest on P1: 2 ± 1% for the cortical ROI and 1 ± 1% for the trabecular ROI. The effect of FOV on failure load was non‐significant, but was largest in GE with on average 4 ± 5% change in failure load.

#### Reconstruction Kernel

The effect of changing the standard reconstruction kernel to a detailed reconstruction kernel was larger than the effects of variations in slice thickness or FOV on the P1, P2, and To scanners (Fig. 4C, Table S‐3). The effect of changing the reconstruction kernel on cortical HU was largest on To, resulting in an average increase of 11 ± 1%. The largest effect on trabecular HU was 16 ± 8% (increase) on To. When the HU were calibrated to BMD, the effects of reconstruction kernel changed, but did not disappear. The effect was largest on P2 (17 ± 1% increase) in the cortical ROI and on P1 (8 ± 3% decrease) in the trabecular ROI. For calculated failure load, the effect of changes in reconstruction kernel were significant on P2 with an average increase of 9 ± 9%, and P1 with an average decrease of 8 ± 5%. The effects of reconstruction kernel were not significant on GE and To.

### Effect of an Air Gap Between Body Model and Calibration Phantom

**Figure 5:**
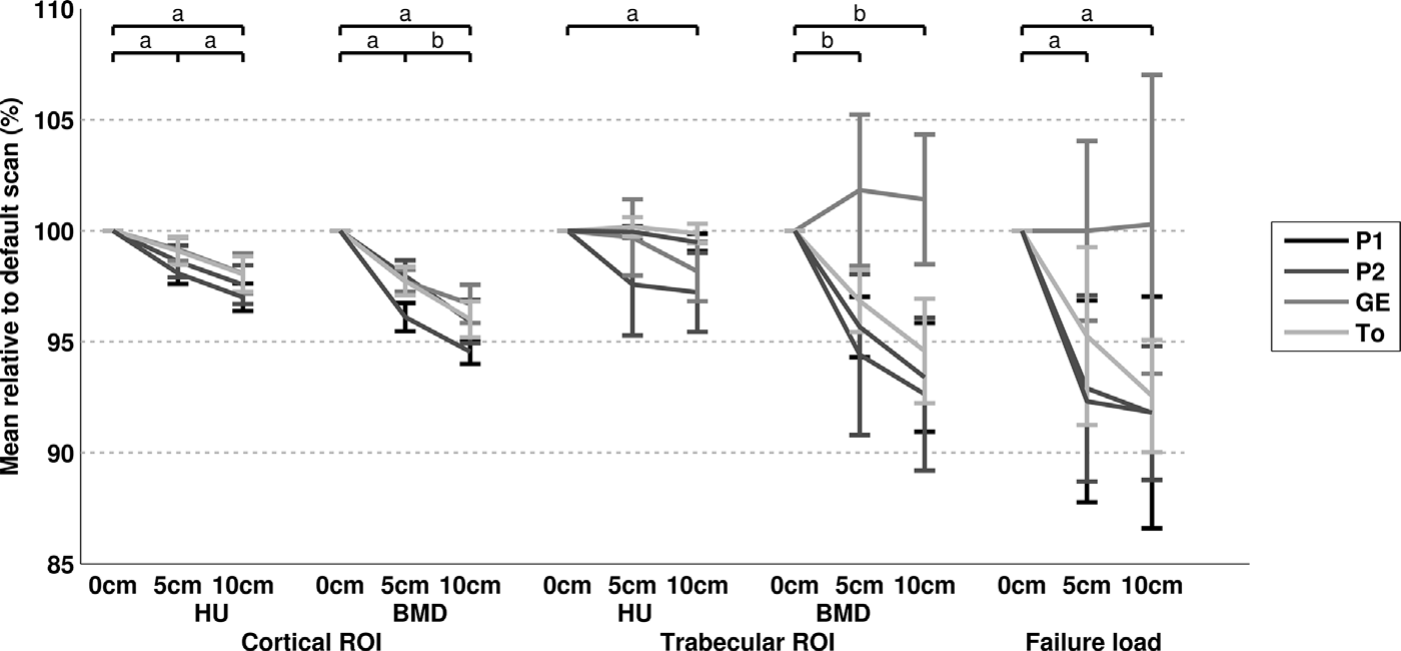
Effect of an air gap (0, 5, and 10 cm) between calibration phantom and body model as percentage (mean ± SD) relative to the standard scan (0 cm air gap, 3 mm slices, FOV 480, standard kernel) for cortical HU and BMD, trabecular HU and BMD, and simulated failure load. a, significant difference on all CT scanners. b, significant difference on P1, P2, and To.

In the cortical ROI, the HU decreased significantly when there was an air gap between calibration phantom and body model (max 3 ± 1% on P1; Fig. 5, Table S‐4). The effect of a 5 cm air gap was not significant in the trabecular ROI (max 2 ± 2% on P2), while a 10 cm air gap resulted in a significant decrease in HU (max 3 ± 2% on P2). The air gap resulted in a decreased cortical BMD on all scanners (max 5 ± 1% on P1), and a decreased trabecular BMD on the P1, P2, and To scanner (max 7 ± 3% on P2). Also, an air gap resulted in a decrease in failure loads (max 8 ± 3% on P2).

## DISCUSSION

This study aimed to quantify the effect of (i) different CT scanners; (ii) different CT protocols (with variations in slice thickness, FOV, and reconstruction kernel); and (iii) air between calibration phantom and patient on HU, BMD, and FE failure load.

We confirmed that differences between scanners in HU, BMD, and calculated failure loads can exist, even when a standard CT protocol is used and scanners are regularly calibrated according to manufacturer's specifications. A recent study by our group, using tissue characterization phantoms, showed that differences between scanners decreased after calibration using a calibration phantom to BMD.^11^ The current study evaluated the inter‐scanner effects using cadaveric femora; however, now, the use of a calibration phantom did not always correct for inter‐scanner differences. Hence, using CT images from various scanners resulted in differences in BMD and subsequent failure loads. The study of Carpenter et al.^13^ determined inter‐scanner differences with the use of femora, and determined BMD in healthy subjects based on QCT images. They reported differences up to ∼20% in cortical and ∼40% in trabecular BMD, when patients were scanned on two different CT scanners. In their case, this led to an average of ∼12% and maximally ∼23% in subsequently calculated failure loads with the use of FE models that simulated single‐leg stance. Percentage‐wise, most of our inter‐scanner differences were smaller than those of Carpenter et al.,^13^ while the differences in both trabecular BMD and failure load between the Toshiba CT scanner and the other scanners were similar to their findings.

The effect of variations in slice thickness and FOV was small (<4% on average), whereas the effect of reconstruction kernel was larger (average of 16% in HU, 17% in BMD, and 9% in failure load). The current HU results were in correspondence with our previous phantom study, also showing small effects of changes in slice thickness and FOV, while reconstruction kernel did affect HU considerably.^11^ In general, the variation in failure load between the femora was larger compared to the variations in HU and BMD. Possibly, this is due to the exponential functions to calculate material properties.^19^ Additionally, the calculated failure load is the result of many numeric calculations that may increase the effect of small deviations in input data. Nevertheless, the exact algorithms behind different reconstruction kernels that calculate the HU from the X‐ray projection data remain unknown. It is, therefore, hard to predict in what way the CT images will be affected by a certain kernel. On different CT scanners, different reconstruction kernels were chosen and each kernel had different effects on the outcomes. In most cases, the calibration phantom was not able to correct the effect of changes in reconstruction kernel. For example, on P1 relatively high effects on HU in the cortical ROI were smaller after calibration to BMD, but the small effect on the trabecular HU increased after calibration on the same scanner. The Toshiba scanner was an exception: the effect of reconstruction kernel on HU was quite large, but was much smaller after calibration to BMD, and as a result, the kernel had a relatively small effect on the calculated failure load. Other studies also found that different reconstruction kernels lead to differences in HU,^9^, ^14^ BMD,^14^ and FE calculated vertebral stiffness.^20^ Calibration with the use of a calibration phantom did not decrease this effect.^14^

As a third aim, we investigated the effect of an air gap between body model and calibration phantom. HU in the cortical and trabecular ROI decreased when the knees of the body model were lifted from the calibration phantom (≤3% on average), which is probably due to changes of the position of the femur in the scanner gantry.^8^, ^11^, ^21^ Since in most cases the effect of the air gap was larger after calibration (≤5% on average), we assumed that the calibration phantom was somewhat affected by the air‐tissue transition, automatically resulting in decreased simulated failure load on all CT scanners (≤8% on average) except for GE. However, to what extent the simulations were affected by the air artifact remains unclear, as the change in position in the gantry also played a role. In our previous patient study,^7^ we noted that air gaps induced a visibly larger artifact in the calibration phantom compared to the current results. Additionally, this artifact was only seen in scans made on a relatively old CT scanner (AcQSim CT, Philips Medical Systems, Eindhoven, The Netherlands). Possibly, newer scanners can better handle the tissue‐air‐phantom transition, although air gaps should be avoided if possible.

Our study had some limitations. First of all, femora were thawed and refrozen multiple times, which might have led to some bone tissue damage. Possibly, this caused the change in bone (marrow) composition in the trabecular bone in one femoral head over time, leading to exclusion of this femur from the analysis of the trabecular ROI. Secondly, the position of the femora was not completely identical in each scanner. Femoral head placement in the acetabulum was comparable, but anteversion angles could deviate between scans. Nevertheless, we chose to use the body model to better resemble an actual patient's CT scan.^16^ Additionally, the body model position could vary somewhat between the CT scanners, despite careful position in the isocenter of each gantry. However, the position variations were very small and, therefore, we do not expect any significant effects of the placement of the femora and body model. We used an automated algorithm for registrations, which error is anticipated to be less than a single voxel.^22^, ^23^ In addition, the registration between a coarser and finer scan can be a source of additional variation.

Ultimately, we aim to correct for CT scanner or protocol related variations. Previously, Keyak et al.^19^ assumed that differences between CT scanners and protocols or other varying parameters could be corrected when using a calibration phantom. Additionally, Giambini et al.^20^ stated that the research community should come up with a standard clinical CT protocol as input for FE models. However, the present study showed that calibration did not always suffice, suggesting that even the use of a standard protocol could not fully correct for inter‐scanner differences. Although we only applied the material behavior as described by Keyak et al.,^19^ we expect that the effects of differences in CT scanner or CT protocol would be rather comparable when using other non‐linear relationships. Therefore, it would be better to develop an effective method for correcting such differences, for example by comparing different kernels beforehand and choosing the most similar kernels between CT scanners for patient scans. In our patient study, we aim to differentiate between high and low fracture risk patients based on failure loads calculated by CT‐based FE models. With respect to the fracture risk predictions, the differences between CT scanners and settings would be critical for patients that have a failure load around the threshold that distinguishes high from low fracture risk patients. In those cases, a patient would switch from a high‐fracture risk prediction to a low‐fracture risk prediction, or the other way around, when scanned on another CT scanner or with another kernel. Based on the results from the current study, we suggest applying a CT scanner‐ and setting‐dependent level of uncertainty to the failure loads of patients’ femora. Subsequently, the patients can be categorized in three groups: high fracture risk, possible high fracture risk, and low fracture risk.

Within the process of creating an FE model, there are many other variables that can result in variations in failure load. Such uncertainties are unwanted when giving patient‐specific advice. Although we now have investigated the effect of CT scanners and protocols, other factors, such as the effect of loading conditions or lytic versus blastic lesions, should be explored as well in the future.

In conclusion, this study showed that quantitative analysis of CT images acquired with different CT scanners could induce changes in HU, BMD, and calculated failure load up to 17%. When using different CT settings, changes in slice thickness and FOV had small effects (≤4% on average), but reconstruction kernels induced variations up to on average 9% in failure load. Additionally, air between patient and calibration phantom slightly decreased the HU, BMD and failure loads (≤8% on average), and should therefore, if possible, be avoided. Finally, for using FE modeling as a clinical tool to predict fracture risk, we suggest applying a CT scanner‐ and setting‐dependent level of uncertainty to the femoral failure load of patients, and categorizing them in three groups: high fracture risk, possible high fracture risk, and low fracture risk.

## AUTHORS’ CONTRIBUTIONS

FE contributed to research design, acquisition, analysis and interpretation of data, and drafting the paper. LCD, YM van der L, NV, and ET contributed to research design, interpretation of data and revising of the paper. JF contributed to research design, data acquisition, and revising of the paper. R van L contributed to research design and revising of the paper. All authors have read and approved the final submitted manuscript.

## Supporting information

Additional supporting information may be found in the online version of this article.

Supporting Table S1.

Supporting Table S2.

Supporting Table S3.

Supporting Table S4.
