# Supplementary material for: Effect of different CT scanners and settings on femoral failure loads calculated by finite element models
Source: J Orthop Res. 2018 Apr 20;36(8):2288–95. doi: 10.1002/jor.23890 (PMC6120464; doi:10.1002/jor.23890)
Supplement: Supplementary file 1 — Supporting Table S1. [file JOR-36-2288-s001.pdf]

Table S-1: Outcomes of the standard scan on each CT scanner for each femur separately. Note that the standard deviations (SD) of the HU are mainly caused by the anatomy of the bone structure within the ROI.

		Femur	P1	P2	GE	To
Cortical ROI	HU	#1	1043 (328)	1032 (358)	1004 (352)	1083 (381)
		#2	1181 (238)	1197 (238)	1158 (234)	1234 (264)
		#3	1095 (125)	1107 (116)	1065 (135)	1162 (157)
		#4	1125 (234)	1131 (228)	1102 (223)	1144 (252)
		#5	1175 (237)	1180 (224)	1134 (247)	1228 (266)
		#6	1290 (114)	1289 (111)	1253 (112)	1341 (130)
	BMD (mg/cm^3)	#1	908	927	874	876
		#2	1028	1075	1009	999
		#3	963	993	932	944
		#4	989	1015	964	930
		#5	1032	1056	992	997
		#6	1133	1154	1097	1089
Trabecular ROI	HU	#1	103 (97)	99 (103)	108 (100)	98 (103)
		#2	318 (139)	311 (152)	315 (141)	317 (148)
		#3	308 (182)	307 (190)	314 (173)	311 (182)
		#4	278 (139)	267 (166)	280 (154)	278 (148)
		#5	130 (131)	126 (139)	140 (130)	132 (130)
	BMD (mg/cm^3)	#1	88	91	91	76
		#2	276	281	272	254
		#3	271	277	272	249
		#4	245	241	242	222
		#5	114	115	119	104
Failure load (N)		#1	2309	2517	2590	2096
		#2	7152	7500	7224	6429
		#3	4778	5170	5315	4543
		#4	6824	6012	6677	5385
		#5	1272	1340	1499	1218
		#6	4283	4140	4244	3815
