# Supplementary material for: Effect of different CT scanners and settings on femoral failure loads calculated by finite element models
Source: J Orthop Res. 2018 Apr 20;36(8):2288–95. doi: 10.1002/jor.23890 (PMC6120464; doi:10.1002/jor.23890)
Supplement: Supplementary file 2 — Supporting Table S2. [file JOR-36-2288-s002.pdf]

Table S-2: Output of the statistical linear mixed models (difference, 95% confidence interval and p-value) of the absolute differences between the CT scanners using the standard protocol (3 mm slices, FOV 480, standard kernel), for HU and BMD in the cortical and trabecular ROI, and simulated failure load (N).

**Cortical HU**			
Scanners	Difference	95% CI	p-value
P1-P2	-9.1	-15.1 – -3.1	0.003
P1-GE	28.5	22.6 – 34.5	<0.001
P1-To	-54.1	-60.1 – -48.1	<0.001
P2-GE	37.6	31.6 – 43.6	<0.001
P2-To	-45.0	-51 – -39	<0.001
GE-To	-82.6	-88.6 – -76.6	<0.001
**Cortical BMD (mg/cm^3)**			
Scanners	Difference	95% CI	p-value
P1-P2	-35.1	-41.7 – -28.5	<0.001
P1-GE	22.9	16.3 – 29.5	<0.001
P1-To	24.2	17.6 – 30.7	<0.001
P2-GE	58.0	51.4 – 64.6	<0.001
P2-To	59.3	52.7 – 65.8	<0.001
GE-To	1.3	-5.3 – 7.8	0.7
**Trabecular HU**			
Scanners	Difference	95% CI	p-value
P1-P2	5.9	3.7 – 8.1	<0.001
P1-GE	-3.3	-5.5 – -1	0.004
P1-To	2.2	0 – 4.4	0.05
P2-GE	-9.2	-11.4 – -6.9	<0.001
P2-To	-3.7	-5.9 – -1.5	0.001
GE-To	5.5	3.2 – 7.7	<0.001
**Trabecular BMD (mg/cm^3)**			
Scanners	Difference	95% CI	p-value
P1-P2	-3.1	-7.7 – 1.6	0.2
P1-GE	-3.4	-8 – 1.3	0.2
P1-To	17.6	13 – 22.2	<0.001
P2-GE	-0.3	-4.9 – 4.3	0. 9
P2-To	20.7	16 – 25.3	<0.001
GE-To	21.0	16.3 – 25.6	<0.001
**Failure load (N)**			
Scanners	Difference	95% CI	p-value
P1-P2	-82.7	-266 – 100.6	0.4
P1-GE	-79.0	-262.4 – 104.3	0.4
P1-To	497.0	313.6 – 680.3	<0.001
P2-GE	3.7	-179.7 – 187	1.0
P2-To	579.7	396.3 – 763	<0.001
GE-To	576.0	392.7 – 759.3	<0.001
