# Supplementary material for: Effect of different CT scanners and settings on femoral failure loads calculated by finite element models
Source: J Orthop Res. 2018 Apr 20;36(8):2288–95. doi: 10.1002/jor.23890 (PMC6120464; doi:10.1002/jor.23890)
Supplement: Supplementary file 3 — Supporting Table S3. [file JOR-36-2288-s003.pdf]

Table S-3: Output of the statistical linear mixed models (difference, 95% confidence interval and p-value) of the absolute effects of changes in Slice thickness, FOV and Reconstruction kernel, for HU and BMD in the cortical and trabecular ROI, and simulated failure load (N). *The interaction between CT scanner and slice thickness or FOV was not significant and therefore, the effects of slice thickness and FOV hold for all of the CT scanners.

**Cortical HU**				
		Effect	95% CI	p-value
Slice thickness*		11.5	8.5 – 14.5	<0.001
FOV*		-10.8	-13.8 – -7.8	<0.001
Kernel	P1	102.9	96.9 – 108.9	<0.001
	P2	-39.7	-45.6 – -33.7	<0.001
	GE	15.6	9.6 – 21.6	<0.001
	To	133.2	127.2 – 139.2	<0.001
**Cortical BMD (mg/cm^3)**				
		Effect	95% CI	p-value
Slice thickness*		11.8	8.5 – 15	<0.001
FOV*		-11.0	-14.3 – -7.7	<0.001
Kernel	P1	25.2	18.6 – 31.7	<0.001
	P2	183.2	176.6 – 189.8	<0.001
	GE	1.2	-5.4 – 7.7	0.7
	To	27.0	20.5 – 33.6	<0.001
**Trabecular HU**				
		Effect	95% CI	p-value
Slice thickness*		-1.2	-2.3 – -0.1	0.04
FOV*		-1.1	-2.2 – 0	0.05
Kernel	P1	2.5	0.2 – 4.7	0.03
	P2	-14.9	-17.1 – -12.6	<0.001
	GE	1.7	-0.6 – 3.9	0.1
	To	29.9	27.7 – 32.1	<0.001
**Trabecular BMD (mg/cm^3)**				
		Effect	95% CI	p-value
Slice thickness*		-1.0	-3.3 – 1.3	0.4
FOV*		-0.7	-3 – 1.7	0.6
Kernel	P1	-14.5	-19.1 – -9.9	<0.001
	P2	14.6	10 – 19.2	<0.001
	GE	-2.5	-7.1 – 2.1	0.3
	To	-12.1	-16.7 – -7.5	<0.001
**Failure load (N)**				
		Effect	95% CI	p-value
Slice thickness*		100.5	8.9 – 192.2	0.03
FOV*		-61.8	-153.5 – 29.8	0.2
Kernel	P1	-312.0	-495.3 – -128.6	0.001
	P2	582.5	399.2 – 765.8	<0.001
	GE	-73.0	-256.4 – 110.3	0.4
	To	-179.8	-363.2 – 3.5	0.06
