# Supplementary material for: Effect of different CT scanners and settings on femoral failure loads calculated by finite element models
Source: J Orthop Res. 2018 Apr 20;36(8):2288–95. doi: 10.1002/jor.23890 (PMC6120464; doi:10.1002/jor.23890)
Supplement: Supplementary file 4 — Supporting Table S4. [file JOR-36-2288-s004.pdf]

Table S-4: Output of the statistical linear mixed models (difference, 95% confidence interval and p-value) of the absolute effects of an air gap (0, 5, 10 cm) between calibration phantom and body model, for HU and BMD in the cortical and trabecular ROI, and simulated failure load (N). *The interaction between CT scanner and air gap was not significant for cortical HU, trabecular HU and failure load and therefore, these effects of slice thickness and FOV hold for all of the CT scanners.

**Cortical HU**									
	0 cm - 5 cm			5 cm - 10 cm			0 cm - 10 cm		
Scanner	Difference	95% CI	p-value	Difference	95% CI	p-value	Difference	95% CI	p-value
All*	-14.2	-20.1 – -8.3	<0.001	-12.2	-18.1 – -6.3	<0.001	-26.4	-32.3 – -20.5	<0.001
**Cortical BMD (mg/cm^3)**									
	0 cm - 5 cm			5 cm - 10 cm			0 cm - 10 cm		
Scanner	Difference	95% CI	p-value	Difference	95% CI	p-value	Difference	95% CI	p-value
P1	-39.0	-49.3 – -28.7	<0.001	-15.7	-25.9 – -5.4	0.003	-54.7	-65 – -44.4	<0.001
P2	-20.7	-31 – -10.5	<0.001	-21.1	-31.4 – -10.9	<0.001	-41.9	-52.1 – -31.6	<0.001
GE	-21.9	-32.2 – -11.7	<0.001	-9.8	-20 – 0.5	0.06	-31.7	-42 – -21.4	<0.001
To	-21.8	-32.1 – -11.5	<0.001	-16.7	-27 – -6.4	0.001	-38.5	-48.8 – -28.2	<0.001
**Trabecular HU**									
	0 cm - 5 cm			5 cm - 10 cm			0 cm - 10 cm		
Scanner	Difference	95% CI	p-value	Difference	95% CI	p-value	Difference	95% CI	p-value
All*	-1.4	-3.2 – 0.4	0.1	-1.3	-3.1 – 0.5	0.2	-2.7	-4.5 – -0.9	0.004
**Trabecular BMD (mg/cm^3)**									
	0 cm - 5 cm			5 cm - 10 cm			0 cm - 10 cm		
Scanner	Difference	95% CI	p-value	Difference	95% CI	p-value	Difference	95% CI	p-value
P1	-7.7	-12.1 – -3.3	0.001	-3.6	-8 – 0.7	0.1	-11.4	-15.7 – -7	<0.001
P2	-9.3	-13.7 – -5	<0.001	-3.4	-7.8 – 0.9	0.1	-12.8	-17.2 – -8.4	<0.001
GE	1.5	-2.9 – 5.9	0.5	-0.4	-4.8 – 4	0.9	1.1	-3.3 – 5.4	0.6
To	-5.0	-9.3 – -0.6	0.03	-3.4	-7.8 – 1	0.1	-8.3	-12.7 – -4	<0.001
**Failure load (N)**									
	0 cm - 5 cm			5 cm - 10 cm			0 cm - 10 cm		
Scanner	Difference	95% CI	p-value	Difference	95% CI	p-value	Difference	95% CI	p-value
All*	-231.3	-354.6 – -107.9	<0.001	-43.9	-167.3 – 79.5	0.5	-275.2	-398.5 – -151.8	<0.001
